# HOW DOES PARENT-CHILD RELATIONSHIP AFFECT CARE? FOCUSING ON MOTHER-DAUGHTER CAREGIVING

**DOI:** 10.1093/geroni/igz038.3273

**Published:** 2019-11-08

**Authors:** Ayako Baba

**Affiliations:** the University of Tokyo, Tokyo, Japan

## Abstract

OBJECTIVE: Long-term caregiver (child)/care-recipient (parent) relationships have both positive and negative effects on care. However, the mechanism of that impact is unclear. This study aimed to explore how parent–child relationships affect care and which aspects cause those effects. METHOD: Five hundred thirty-four adult children who were caring for or had cared for their parents at home completed the scales of parent–child psychological independence, the acceptance of care, care attitude, and care burden. Data were analyzed using a pass analysis with multiple group structural equation modeling to identify the relationship between parent–child psychological independence, acceptance of care, care attitude, and care burden, and the care dyad difference of the models. RESULT: 1) “Reliable relationship with parent” in parent–child psychological independence affected “resignation” and “understanding actively” in acceptance of care. 2) “Psychological individuation from the parent” in parent–child independence affected all subscales of care attitudes. 3) “Resistance” and “understanding actively” in acceptance of care and “auto-pilot” in care attitude affected care burden. 4) In mother–daughter caregiving, “resistance” and “resignation” had stronger effects on “auto-pilot” whereas “utilization of resource” and “flexible response” in care attitude and “resistance” had weaker effects on care burden. CONCLUSION: The relationship between long-term parent–child relationship and care were revealed. In some points, daughters who were caring for or had cared for their mothers had a different model from other care dyads. These results suggest that child caregivers should be supported mentally in accordance to their difficult points and dyads.

